# Effects of body visualization on performance in head-mounted display virtual reality

**DOI:** 10.1371/journal.pone.0239226

**Published:** 2020-09-21

**Authors:** Stefan Pastel, Chien-Hsi Chen, Katharina Petri, Kerstin Witte

**Affiliations:** Department of Sports Engineering and Movement Science, Otto-von-Guericke-University Magdeburg, Institute III: Sports Science, Magdeburg, Germany; Manchester Metropolitan University - Cheshire Campus, UNITED KINGDOM

## Abstract

Although there are many virtual reality (VR) applications in sports, only a handful of studies visualized the whole body. There is still a lack of understanding, how much of the own body must be visualized in the head-mounted display (HMD) based VR, to ensure fidelity and similar performance outcome as in the real-world. In the current study, 20 young and healthy participants completed three tasks in a real and virtual environment: balance task, grasping task, and throwing task with a ball. The aim was to find out the meaning of the visualization of different body parts for the quality of movement execution and to derive future guidelines for virtual body presentation. In addition, a comparison of human performance between reality and VR, with whole-body visualization was made. Focusing on the main goal of the current study, there were differences within the measured parameters due to the visualization of different body parts. In the balance task, the differences within the VR body visualization consisted mainly through no-body visualization (NB) compared to the other visualization types defined as whole-body (WB), WB except feet (NF), as well as WB except feet and legs (NLF). In the grasping task, the different body visualization seemed to have no impact on the participants’ performances. In the throwing task, the whole-body visualization led to higher accuracy compared to the other visualization types. Regarding the comparison between the conditions, we found significant differences between reality and VR, which had a large effect on the parameters time for completion in the balance and grasping task, the number of foot strikes on the beam in the balance task, as well as the subjective estimation of the difficulty for all tasks. However, the number of errors and the quality of the performances did not differ significantly. The current study was the first study comparing sports-related tasks in VR and reality with further manipulations (occlusions of body parts) of the virtual body. For studies analyzing perception and sports performance or for VR sports interventions, we recommend the visualization of the whole body in real-time.

## Introduction

There are many different applications of a head-mounted display (HMD) based virtual reality (VR), but only a few studies integrated a virtual body in the context of sports applications (for review see [1]). Furthermore, VR is a promising tool for expanding the possibilities of psychological and sport training applications due to many aspects in the virtual environment that can be controlled and manipulated, which are not possible in a real-world setting [2]. VR allows for the manipulation of the body representation in terms of structure, size, morphology, and perspective [3, 4]. Skills learned in adequate VR training with proper stimuli can be transferred to a real-world setting (for review see [5]). Previous studies in the field of psychology have shown that a virtual body can increase the feeling of presence and the degree of reality (e.g. [6]), especially when participants are allowed to choose a favorite design [7], and when the virtual body is realistic [8].

Fidelity, that means a simulation, which recreates the real-world system, leads to the users’ feel of presence in VR [9]. The authors also described the confusion about terms like fidelity, validity, immersion, and presence and gave a good overview of how to use them in the right context. They also presented different types of fidelity for example the physical one, which refers to the level of realism provided by the simulation. They stated that physical fidelity is important to elicit a feeling of the presence of the participant [9]. An easy and valid approach to assess the strength of the feeling of ‘being there’ is to use questionnaires (for example see [10, 11]), which used Likert scales to gauge the participants’ impression [12]. A further recommendation for testing the presence is to measure physiological responses and behavior for example the stress level measured by increasing skin conductance responses [12]. The sensation of presence, as a psychological, attentional, and cognitive state, is linked to psychological and contextual factors, cognitive and sensorimotor aspects, as well as to visuo-proprioceptive coherency [13]. Therefore, not only a virtual body is needed, but a body, which is accepted by the users. Experiences with the own body also have to match experiences with the virtual body. It is evident, that the body schema is changeable due to the neuroplasticity in the human brain [14]. Good body-ownership (when participants accept and can control the virtual body) also leads to better accuracy in movement tasks [15]. The virtualization of the own body can further be important for distance estimation [16], grasping tasks [17], as well as for the improvement of action control, performance accuracy, and lower limb coordination during obstacle avoidance [18]. For a review of distance estimation in VR, see [19], as well as [20] which showed that different results depend on different measuring methods. However, [13] found that the integration of haptic (vibrotactile) feedback further increases performance in navigation and grasping tasks, and it supports the correct position of virtual body segments by using visual information.

Virtual bodies can be accepted very quickly by the users, and can also be easily controlled, even if these bodies do not match the own body shape or body size, or if the virtual body contains additional body parts (e.g. a tail or [21]). In a review with a focus on rehabilitation, [22] explain the importance of movement visualization for learning progress in VR interventions. In recent reviews, [3] discussed the sense of embodiment, and [23] explained principles of bodily self-consciousness based on recent experiments about imagined body-ownership and rubber hand experiments. Virtual bodies that are different from the own real body (e.g. a body from a child [24]), or from an ethnic minority [25] can also be accepted quickly, and users change their behavior according to the body size, body shape and the social role. Virtual bodies can, therefore, affect perception and behavior [8].

Virtual embodiment is crucial for controlling the VR and communicating with the environment or other virtual characters. [26] demonstrated that humans respected the same behavioral rules (e.g. interpersonal distance) in VR as in the real-world. As well as other behavioral patterns were observed, e.g. men who were shy around women in the real-world were also shy around female virtual characters [26]. However, it is still unknown if humans accept virtual characters as presented humanoid, and if there are any differences in interpersonal behavior between several kinds of characters, e.g. agents controlled by a computer or agents controlled by an other human [27].

At the present time, only a few numbers of studies included the whole-body visualization. In most studies, either nothing of the own body or only some body parts (in most cases the hands for better orientation) were visualized [1]. So far, whole body visualizations in HMD based VR were used for therapy [7] or for investigations of embodiment to analyze the link between central body representations and higher cognitive functions [14, 23]. In sports, the whole body (or body-part) visualizations were utilized to increase the degree of realism, to support spatial navigation, and to decrease symptoms of cybersickness, which can be defined as physical discomfort elicited by the stay in VR. [28] used whole body visualizations in squat movements to examine the influence of such a visualization, as wells as different perspectives on the own motor execution using a virtual mirror. For a review of whole-body motion reconstructions in HMD based VR, we refer to the review of [8]. The tracking of the body in VR applications is an increasing research interest, and for whole-body tracking marker-based, marker-less (in most cases kinect systems), as well as inertial measurement units can be utilized [8]. Whole-body motion tracking in HMD based virtual environments was shown to be beneficial for spatial presence and involvement [29]. Presenting a virtual body in combination with the head-mounted display based virtual reality is crucial to the sense of being in a virtual environment [12].

Generally, one fundamental research question was: to what extent can virtual bodies be perceived as own bodies in a virtual environment [30]. [3] gave an overview of the meaning of embodiment in a virtual environment. The authors emphasized that embodiment is associated with concepts of the sense of self-location, the sense of agency, and the sense of body ownership. Self-location describes the feeling that one feels self-located inside the biological or an avatar´s body. For this, the first-person perspective is crucial, since the feeling of being self-located can be influenced by the origin of the visuospatial-perspective [31, 32]. The sense of agency is present in active movements and results from the predicted sensory consequences of one’s actions from the efference copy and the actual sensory consequences [3]. The authors also described the sense of body ownership, which implies that the body is the source of the experienced sensations through a combination of bottom-up and top-down processes. Comparable performances of the participants in a real and virtual environment could be a sign of acceptance of the virtual body as their own. Nevertheless, there is still a lack of understanding in how much of the own body must be visualized in HMD based VR to ensure fidelity and similar performance outcome as in the real-world. This could be important for future training recommendations. There already exist some intervention studies in immersive VR, which showed benefits from such training (e.g. [33–35]) but the transfer into reality is often unresolved (for review see [5]). In addition, there are only a few studies, which compared sports-specific behavior in VR and the real-world (e.g. [35, 36]), for review of ball sports in VR, see [37]. While in karate specific studies, no or only slight differences were found between VR and reality, previous results showed that perception, in general, might be different between both conditions due to different usage of the ventral and dorsal stream for visual information processing [2].

There are only a few studies available that compared the motor behavior between VR and reality. Furthermore, in none of these studies, the influence of different body visualization by occlusion of different body parts was analyzed before. According to [8], there is a further need for whole-body motion reconstruction studies and studies that manipulated such whole-body visualizations. Especially, sports specific behavior under different body visualizations is rarely investigated. Therefore, the aim of the current study is to manipulate the presentation of the own body and to investigate the performance in virtual reality compared to reality in sports-related topics, such as balancing on a beam, as well as grasping and throwing a ball for young adults. We used the first-person viewpoint for all tasks. [38] showed that body ownership and embodiment can differ according to the viewpoint. The feeling of presence and embodiment are higher in first-person view [39] but third-person view can be more suitable for novices in motor learning [40]. Furthermore, first-person view is closer to reality than third-person view (side view).

For the development of a sports training scenario in VR, that will be accessible to every interested user, it is important to find out which parts of the body should be visualized in the training scenarios so that adequate training can take place. In addition, a high-developed VR scene was often associated with realistic and detailed properties, but not with it´s functionality in the context of VR training or movement execution [41]. For practical issues, the controllers of the VR-application were often used for visualization of the arms or fists. Having a motion capturing system, which enables the whole-body visualization in real-time is not conceivable for private uses. For sport-related task completion, it should be examined how much of the own body must be perceived. Therefore, the aim of the current study was to find out the meaning of the visualization of different body parts for the quality of movement execution and to derive future guidelines for virtual body presentation. Therefore, young and healthy participants complete three tasks in both conditions: balance task, grasping task, and throwing task. The tasks were chosen because they differ in the needed abilities to complete them and we did not want to specialize in just one. VR can be seen as a useful tool to study human behavior and to examine the impact of body visualization on sports performances since the visual system plays a decisive role during experiences in virtual environments [9]. With less visible properties of the own virtual body, we assume that decreased embodiment leads to significantly worse performances in sport motoric tasks. For this, we manipulated the presentation of the virtual body by occlusion of different body parts and partly manipulated objects and targets (a balance beam, a ball, and a ball cart). Based on previous literature highlighting the importance of the body presentation, we expect that the occlusions of the virtual body will lead to a decrease in performance. Besides, we also analyzed and compared human performance between reality and VR in order to detect possible differences between the conditions. Due to the creation of a very realistic virtual environment and the freedom of natural movements, we expect no differences in performance between reality and VR for the condition presenting the whole body.

## Methods

### Participants

20 healthy students (13 male, 7 female, age: 21.6 ± 1.6 years) with normal or corrected-to-normal vision participated on a voluntary basis. All participants were informed about the aim and procedures and gave their written consent. The approval of the Ethics Committee of the Otto-von-Guericke University at the Medical Faculty and University Hospital Magdeburg was obtained under the number 132/16. A self-made questionnaire assessed that the majority of the participants had a great interest and an open mindedness regarding new technology and VR during research. Therefore, a scale was used with 1 point (does not apply) to 5 points (does apply). The results showed great curiosity of the participants (scale: 4.8 ± 0.4). Besides, 81% of the participants stated pre-experiences regarding the participation of VR-studies.

### Motion capturing and visualization in VR

To realize the whole-body visualization in the VR, the participants wore a black motion suit on which 53 markers were attached, including 5 markers attached to the HMD for the head-tracking. During the data collection of this study, finger tracking was not yet available. The marker setup followed the instruction recommended by the supplier (Vicon, Shogun, Oxford, UK,). Meanwhile, the objects were modeled, tracked and visualized in the same VR scene. These objects included a balance beam, a ball, a green pad, a chair, a small football goal in a ball cart.

To visualize the real-time human movement and the tracked objects in VR, two desktop PCs (source-PC and target-PC) were used and they were connected directly via an internet cable to ensure the high stability and performance of data transmission. The source-PC (equipped with Intel i7 CPU, 32 GB memory, 512 GB SSD, and Nvidia Quadro K2200 4GB graphics card) was running the motion capture system (Vicon, England) with 13 infrared cameras at the sampling rate of 120 Hz. The software (Vicon Shogun, Vicon, England) for capturing the whole-body movement was used to stream the motion data to the target-PC. The target-PC (equipped with Intel i7 CPU, 16 GB memory, 512 GB SSD, and Nvidia GTX 1080 8GB graphics card) was running a self-modeled scene in Unity3D (version 2019.2.11f) and received the live data from the source-PC to drive the movement of the avatar in the VR. SteamVR Plugin for Unity (version 2.5.0) was used to enable all VR functions.

### Procedure

**Fig 1:**
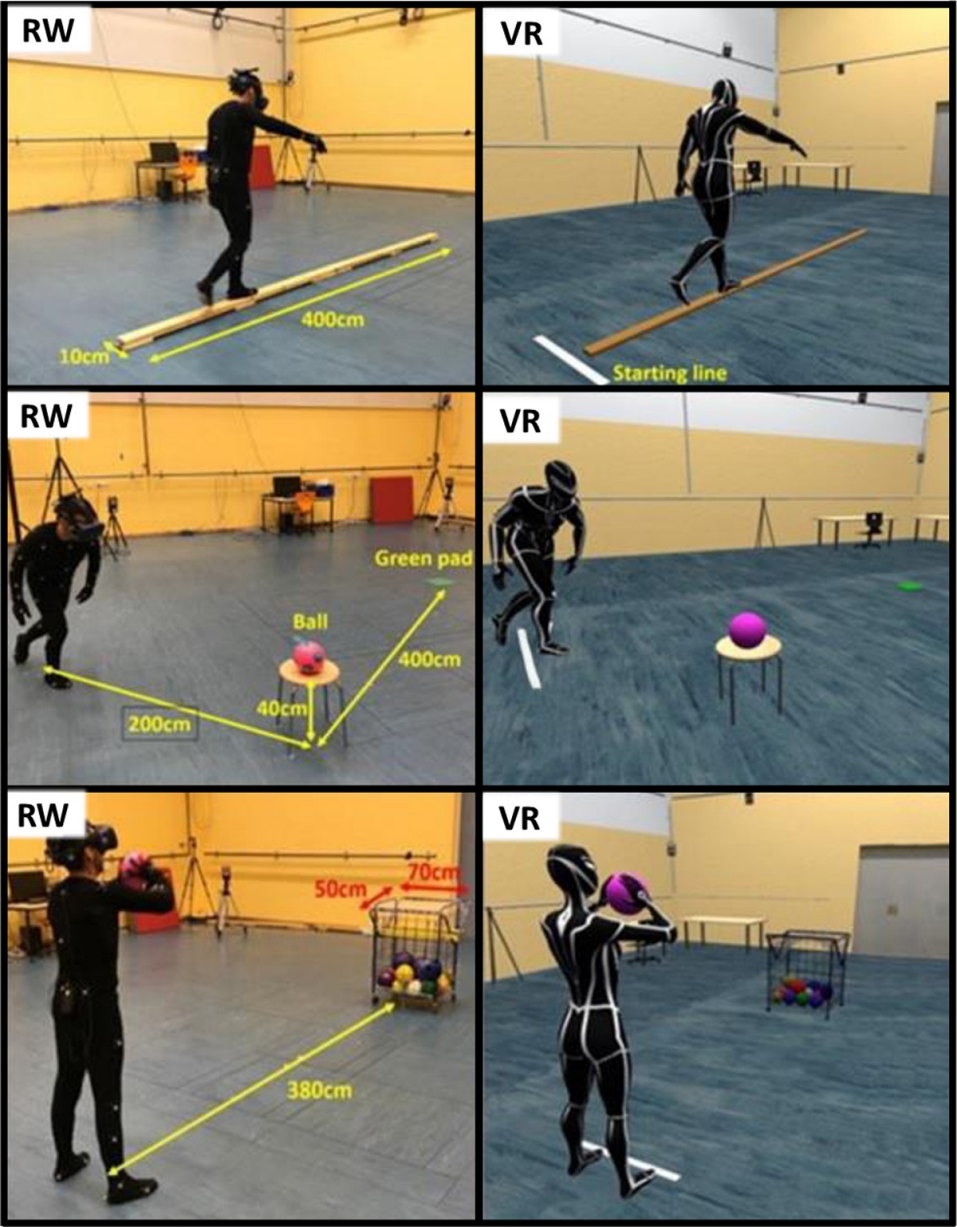
Overview about the different motoric tasks. Balance task (upper panel), grasping task (middle panel) and throwing task (bottom panel) in real-world (RW) and virtual reality (VR). The same order of magnitude was created in both conditions.

All participants first conducted three tasks in VR using a head-mounted display (HMD, HTC Pro Eye, with a total resolution of 2880 x 1600 Pixel, and a field of view of 110 degrees), and afterward, they repeated the tasks in real-world (RW). We chose this order because we assumed that it is easier to perform in RW compared to VR. Moreover, we wanted to provide a more comfortable procedure. In each condition, they started with the balance task, followed by the grasping task, ending with the throwing task. The three tasks are presented in Fig 1.

Four different body visualization conditions were applied in randomized order, and each condition was performed three times in a single task. Thus, 12 repetitions were performed in VR for each task and only three repetitions per task were conducted in RW since the body visualization condition could not be changed. Within every task, we obtained different parameters to assess the sports performance (which is described later). However, it is possible that no differences between the conditions (VR and RW) can occur especially between the different presentations of the virtual body at the expense of a greater cognitive load (e.g. participants need to concentrate more on the tasks). Therefore, immediately after completion of every single trial in every task, we also asked the participants how difficult they rate each trial and we noted these verbal reports. To measure the estimation of difficulty, we used a scale from 0 points (no subjective difficulty) to 10 points (very difficult). Independent of the performances, we were interested in the subjective estimated difficulty of all tasks, since being in an artificial world could lead to the impression of unfamiliarity or discomfort.

**Fig 2:**
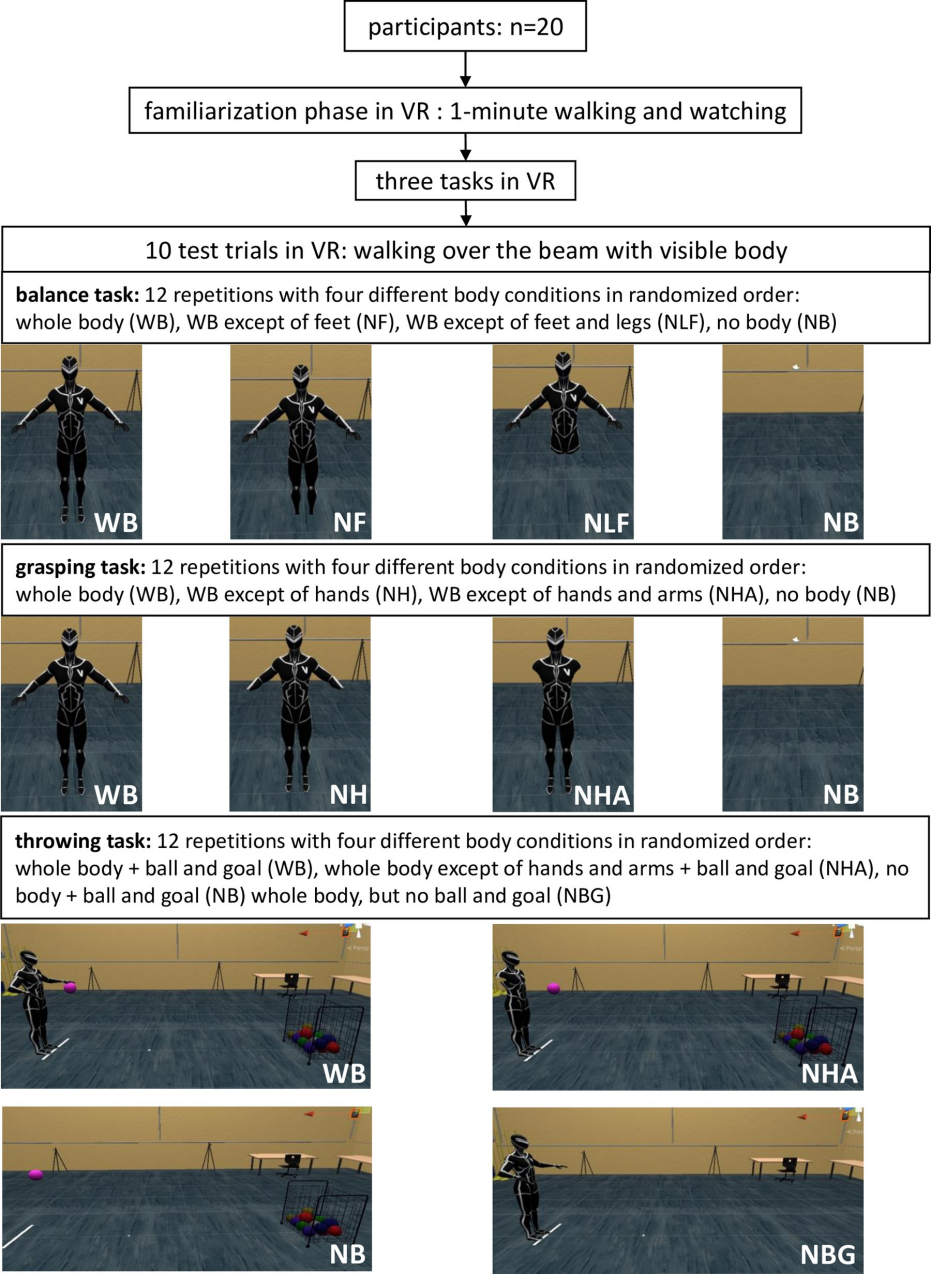
Procedure of the study related to VR. This figure shows the different body part visualizations. The visualization of the whole scene including all visualized objects is given in Fig 1.

The procedure in VR is given in Fig 2. The total duration of the experiment per participant lasted 2 hours and the duration inside VR was around 30 to 40 minutes. Prior to the beginning of the tasks, the participants were free to move around in the virtual environment for 1 minute to get familiar with it. After the tasks in VR were done, the participants had one minute to adjust and recover their visual perception before starting the tasks in RW. This prevents a further time delay and an additional factor of harming participants’ patience. After procedures in VR, the participants conducted the same tasks in RW. As well as in VR, they started with a 1-minute familiarization phase. For all tasks, three trials for each task including only one visualization, whole-body visualization (WB), were conducted by the participants.

#### Balance task

For the balance task, the participants were instructed to walk forward as fast and as accurately as possible over a balance beam laying on the ground with a width of 10 cm, a height of 4 cm and a length of 4m. The participants waited at the starting line in front of the beam (Fig 1), should then walk across it until the end and step down on the ground with one foot after the other. For familiarization in VR, the participants should perform ten trials with whole-body visualization. The parameters, which were obtained to analyze the performance, were the time for completion (time from a verbal “Go”-signal until both feet of the participants touched the ground at the end of the beam), the number of errors (reflects the number when the ground has been touched with one or both legs), and the number of foot strikes on the beam. The different body visualizations are presented in Fig 2.

#### Grasping task

The participants were instructed to start on a given start point (a virtual line on the floor), then go to a ball, grasp it, and put it carefully in a given target area (a green pad 30x30cm) on the floor. In this case, carefully means that the ball must not roll away from the target area. The total distance between the start point and the target area was always 6m. However, the location and the height of the ball changed (either the ball lay on the floor or a chair with a height of 50 cm. We chose these variations to ensure some variation and not to make the task too easy. Again, the completion of the task should be done as fast and as accurately as possible. As parameters served the time for completion (time from a verbal “Go”-signal until the ball hit the target area), and the quality using a scoring system (0 points: grasping and dropping the ball on the target area failed, 1 point: only grasping or dropping of the ball worked, 2: points: grasping and dropping of the ball was performed properly). The body visualizations in VR were whole body (WB), no hands (NH), no hands and arms (NHA), as well as no body (NB) (Fig 2).

#### Throwing task

The participants should throw a ball with both hands (like a chest throw in basketball) into a goal (50 x 70 cm) with a distance of 3.80 m and a height of approximately 1m. According to a scoring system, we analyzed, if and how the ball landed in the goal or not (0 points: ball did not touch the goal, 1 point: ball touched the bars but not the net, 2 points: ball touched the net, see Fig 1). The net was not visualized in VR. As body visualizations, we provided the whole-body (WB), no hands and arms (NHA), as well as no-body (NB) (Fig 2).

### Data analysis

45 trials (36 trials in VR with 12 per task, and 9 trials in RW with three per task) recorded for each participant, thus 900 datasets were obtained with no dropout. All tasks were filmed using a camera (GoPro Hero 6, 60 Hz). The videos were analyzed with the Windows Media Player (Microsoft, Redmond, USA, version 12.0.18362.418) and Magix Video Deluxe Premium (Magix software GmbH Berlin, Germany) to determine the different parameter.

For each performance parameter (time for completion, number of errors, number of the foot strikes in the balance task, and quality in the grasping task and throwing tasks), as well as for the subjective difficulty, Friedman tests were applied with body visualization as a within-subject variable. When the data featured the requirements (no outliers, normal distribution, and sphericity), one-factor variance analysis with repeated measurement ANOVA was conducted. For each task and parameter, we compared four different body visualizations (every four conditions in VR). Furthermore, Dunn-Bonferroni-post-hoc-tests with an estimation of effect sizes were carried out. Effect sizes were obtained using Pearsons’ correlation coefficient (r) being defined as r = 0.1 small effect, r = 0.3 moderate effect, and r = 0.5 large effect. The level of significance was set to α = 0.05. All analyses were carried out with SPSS, version 25. Depending on the data type, we used either t-Tests for dependent samples or Wilcoxon tests to reveal possible differences between RW and VR (WB). In some cases, the number of participants is reduced due to the appearance of significant outliers, which were detected using boxplots graphs.

## Results

Concerning the aims of the study, the results were divided into two parts. The first step was to focus on the comparison of the participants’ accomplishments between the two conditions RW and VR. The results of each parameter and each task are provided in Table 1. For the comparison between the conditions, we considered the whole-body visualization (WB) in condition VR. The main part of the results constitutes the influence of the different types of body visualization in VR, which is shown in Table 2.

**Table 1:** Results of the comparison between real-world (RW) and virtual reality (VR/WB) for the three tasks.

Parameter	RW	VR / WB	two-tailed		effect sizes (Pearson’s r)
			Z	p	
**Balance task**					
time for completion (s) (n = 19)	4.21 ± 0.95	5.93 ± 1.57	-3.783	.000	r = 0.60, large effect
number of foot strikes on the beam (n = 20)	8.52 ± 1.27	10.03 ± 1.37	6.630	.000	r = 0.83, large effect
number of errors (n = 18)	0.07 ± 0.24	0.24 ± 0.42	-1.549	.121	-
subjective estimation of difficulty (1: easy -10: very difficult) (n = 20)	2.15 ± 1.14	3.58 ± 1.43	-3.397	.001	r = 0.54, large effect
**Grasping task**					
time for completion (s) (n = 19)	2.98 ± 0.27	3.78 ± 0.36	11.964	.000	r = 0.94, large effect
quality due to score system (0: bad-2: very good) (n = 20)	1.82 ± 0.28	1.70 ± 0.21	-1.507	.132	-
subjective estimation of difficulty (1: easy -10: very difficult) (n = 20)	1.48 ± 0.70	2.65 ± 1.35	-3.556	.000	r = 0.56, large effect
**Throwing task**					
quality due to score system (0: bad-2: very good) (n = 20)	1.75 ± 0.36	1.68 ± 0.55	-.241	.81	-
subjective estimation of difficulty (1: easy -10: very difficult) (n = 20)	2.03 ± 1.01	2.98 ± 1.37	-3.089	.002	r = 0.48, moderate effect

Mean ± SD are given for each condition. Significant differences between each condition within each parameter are provided using Wilcoxon tests or t-Tests. The estimation of effect sizes (Pearson’s correlation coefficient r) is given. Only the whole-body visualization (WB) in VR was used for the comparison to RW-performances. The number of participants is given by n.

**Table 2:** Results of the three tasks.

**Balance task**						
**Parameter**	**WB**	**NF**	**NLF**	**NB**	**Significance between the body conditions using Friedman tests/ANOVA**	**Dunn-Bonferroni-post-hoc-tests and effect sizes (Pearson’s r) for significant differences**
time for completion (s) (n = 19)	5.93 ± 1.57	5.80 ± 1.05	5.79 ± 1.41	6.82 ± 1.86	χ^2^ (3) = 19.863, p<0.001	NF-NB: p<0.001, r = 0.84 (large effect)
						NLF-NB: p<0.001, r = 0.89 (large effect)
						WB-NB: p<0.001, r = 0.75 (large effect)
number of foot strikes on the beam (n = 20)	10.03 ± 1.37	10.03 ± 1.33	10.10 ± 1.60	10.98 ± 2.10	χ^2^ (3) = 21.313, p<0.001	NF-NB: p<0.001, r = 0.82 (large effect)
						NLF-NB: p<0.001, r = 0.80 (large effect)
						WB-NB: p<0.001, r = 0.81 (large effect)
number of errors (n = 18)	0.24 ± 0.42	0.20 ± 0.26	0.09 ± 0.15	0.28 ± 0.47	χ^2^ (3) = 2.767, p = 0.429	-
subjective estimation of difficulty (1: easy -10: very difficult) (n = 20)	3.58 ± 1.43	3.65 ± 1.82	3.48 ± 1.80	3.98 ± 1.98	*F* (3, 57) = 1.607, p = 0.198	-
**Grasping task**						
**Parameter**	**WB**	**NH**	**NHA**	**NB**	**Significance between the body conditions using Friedman tests**	**Dunn-Bonferroni-post-hoc-tests and effect sizes (Pearson’s r) for significant differences**
time for completion (s) (n = 19)	3.78 ± 0.36	3.80 ± 0.54	3.79 ± 0.45	3.65 ± 0.50	*F* (3, 54) = 1.528, p = 0.218	-
quality due to score system (0: bad-2: very good) (n = 20)	1.70 ± 0.21	1.77 ± 0.22	1.73 ± 0.28	1.87 ± 0.17	χ^2^ (3) = 6.959, p = 0.073	-
subjective estimation of difficulty (1: easy -10: very difficult) (n = 20)	2.65 ± 1.35	3.03 ± 1.73	3.17 ± 1.85	3.15 ± 2.10	χ^2^ (3) = 4.842, p = 0.184	-
**Throwing task**											
**Parameter**	**WB**	**NHA**	**NB**	**Significance between the body conditions using Friedman tests**		**Dunn-Bonferroni-post-hoc-tests and effect sizes (Pearson’s r) for significant differences**
quality due to score system (0: bad-2: very good) (n = 17)	1.68 ± 0.55	1.42 ± 0.52	1.32 ± 0.59	χ^2^ (3) = 13.176, p<0.05		WB-NHA: p<0.05, r = 0.24 (small effect)
						WB-NB: p<0.05, r = 0.34 (moderate effect)
subjective estimation of difficulty (1: easy -10: very difficult) (n = 20)	2.98 ± 1.37	3.55 ± 1.67	3.47 ± 1.81	χ^2^ (3) = 37.153, p<0.001		WB-NB: p<0.05, r = 0.20 (small effect)
						WB-NHA: p<0.05, r = 0.29 (small effect)

Mean ± SD are given for each body visualization condition. Significant differences between each condition within each parameter are provided using Friedman tests or ANOVA. Significant post-hoc-tests and estimation of effect sizes (Pearson’s correlation coefficient r) are given. Body visualization conditions: WB: whole body condition, NF: no feet, NH: no hands, NLF: no feet and no leg, NHA: no hand and arms, NB: no body. The number of participants is given by n.

Most significant differences occurred between RW and VR(WB) but not within the different VR conditions. The participants performed significantly better in reality compared to VR(WB). In reality, they had shorter times for completion, fewer foot strikes on the beam, and the subjective estimation of difficulty were perceived lower in reality compared to VR(WB). However, no significant differences were detected between RW and VR(WB) in the number of errors during balancing as well as in the quality of throwing and grasping.

We found significant differences between the different types of body visualization, which had a large effect for the parameters “time for completion” and “number of foot strikes on the beam” in the balance task, as well as small effects in the “quality of throwing due to score system” and “subjective estimation of difficulty” in the throwing task. The differences within the VR body visualization consist mainly through no-body visualization (NB) compared to the others (see Table 2). Of course, a moderate effect on the quality of throwing and subjective estimation of difficulty is observable when the goal and ball (NBG) were hidden in the participant’s view (see Table 2).

The following graphs give an overview of the performances of the participants within each body visualization type.

## Discussion

Due to the ever-increasing computing power and the representations of realistic-looking virtual scenes, we first examined whether the behavior (in this case the performances of participants) was comparable to that from RW. We found significant differences between VR compared to reality in all three tasks: balancing, grasping a ball from different height and laying it on a target in different distances, and throwing a ball into a target (ball cart) with a chest pass. Performance in reality was better compared to VR. Especially, the time factor differs with large effects between both conditions. The participants stated of having an uncomfortable feeling to complete the balance task and recognized more instability in their performances. The participants were not used to seeing their environment through computer graphics, which could be an explanation for insecurity. However, the goal of each task was achieved with no significant differences in the number of errors (balance task), grasping, and throwing with no significant differences in quality. The subjective estimation of difficulty was higher in VR compared to RW in all three tasks, and the way to reach the goal (significant difference in the number of foot strikes on the beam and time for completion in the balance task) was partly different. Concerning the number of errors, the result is in line with previous work, which found that similar sports performance could be attained in reality and VR (for handball see [42] and for karate see [35]). However, similar performance can be reached on the expanse of different motor execution [42], which can also be dependent on the level of graphical detail [43].

Several studies in VR exist, which analyzed static balance (e.g. [44]), or dynamic balance using force plates (e.g. [45]). In therapy, and especially with older adults, it was found that VR balance interventions had more benefits than conventional balance training (e.g. [46]). However, such studies compared reality and exergames (Desktop VR) [47], and not immersive HMD based VR. In the current study, we examined dynamic balance when comparing balancing over a balance beam in VR with reality. The performance was worse in VR compared to reality because significantly more time was needed and more foot strikes were taken to complete the task. However, It should be emphasized that on average, the difference in the numbers of foot strikes between RW and VR was quite low, and therefore, a small amount of time delay occurred (see Table 1). Although the properties in the current virtual scene were identical to those from the real environment, the development of higher sophisticated realistic-looking virtual scenes through increased computing power could reduce those minimal differences. Nevertheless, the participants’ subjective impression of the virtual room was good. This was confirmed through their given feedback, in which 75% of the participants perceived the virtual room as realistic or quite similar to the real environment. Just 5% of the participants were focused on the fun factor, and the remaining percent stated unfamiliarity and blurriness.

Many ball sports in VR examined interception of balls, such as anticipating landing points and analyzing the influence of ball spins. However, only a few studies investigated throwing in VR so far (for review see [37]). [40] found that learning free throws in a CAVE is best when beginners see their performance from third-person perspective and with additional ball flight information (ball trajectories). However, that scenario is not realistic with learning in reality, therefore, we decided to perform each task with first-person perspective. [48] analyzed throwing with different distances and found that with increasing distance to the target, the performance decreases. Therefore, we chose a quite close distance. Compared to WB condition, the different types of visualization, the NHA, and NB differ in the quality as well as in the subjective estimation of difficulty. That difference showed that for throwing quality, it is easier when the whole body is visible all the time. The occlusion of the body parts probably harmed memorizing the target position and its’ properties.

**Fig 3:**
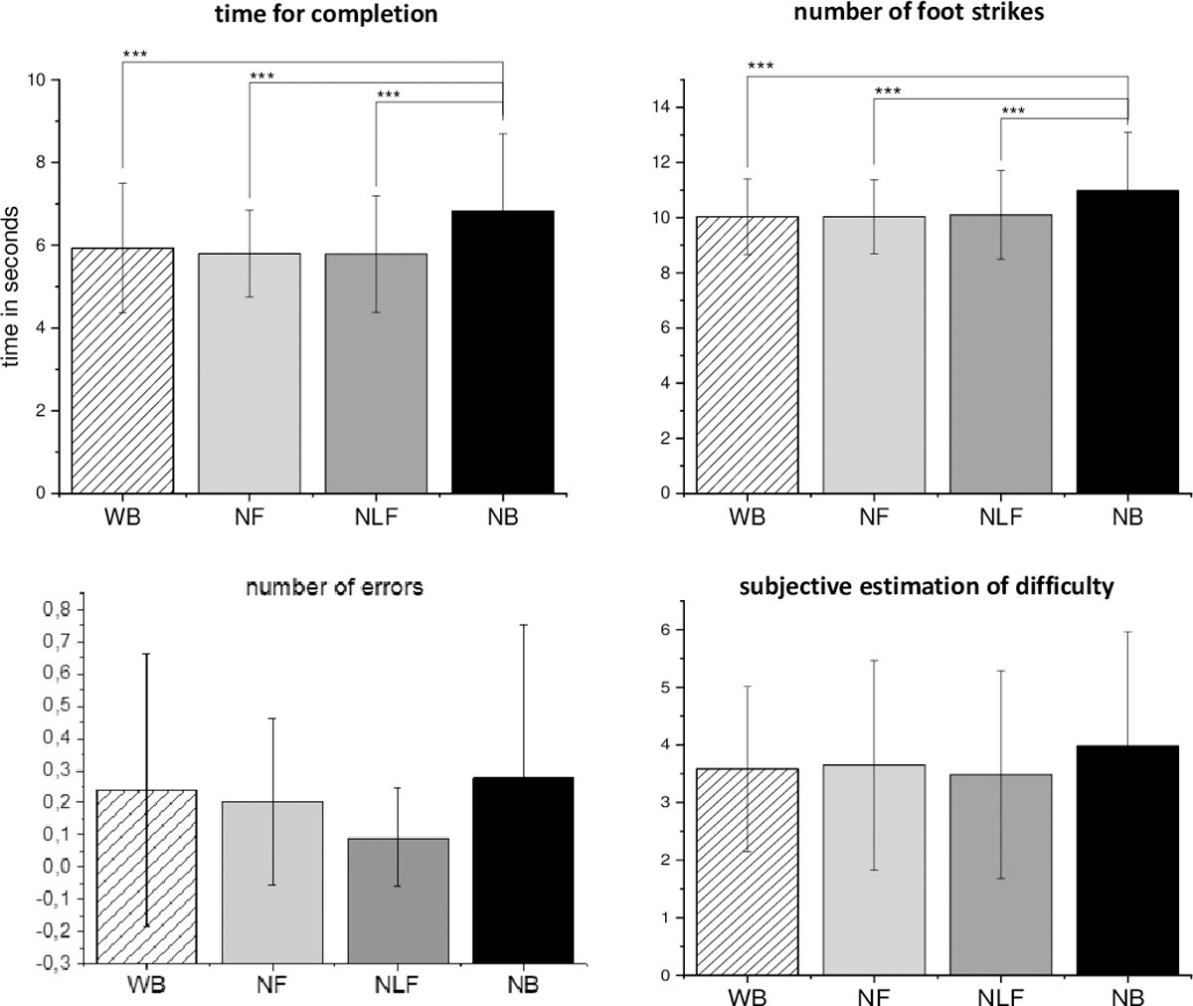
Overview of every parameter for the balance task. Black bar indicates the value for whole-body (WB), the grey bar for no leg and feet, the blue bar for no feet and the red bar for the no-body visualization. The probability of error is indicated through * = p≤0.05; ** = p≤0.01; *** p≤0.001.

**Fig 4:**
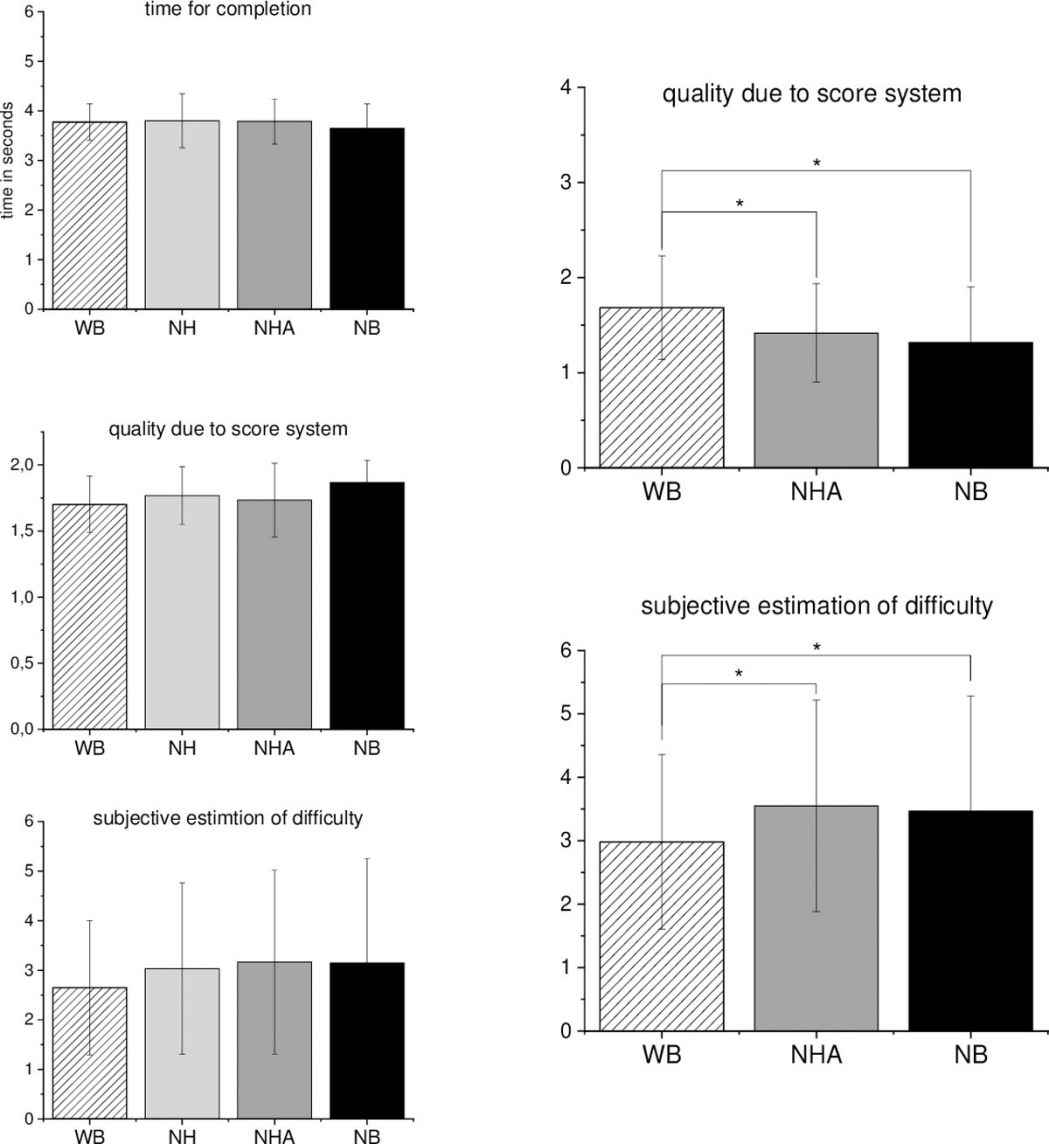
Overview of every parameter for the grasping and throwing task. Black bar indicates the value for whole-body (WB), the grey bar for no leg and feet, the blue bar for no feet and the red bar for the no-body visualization. The probability of error is indicated through * = p≤0.05; ** = p≤0.01; *** p≤0.001.

The main goal was to analyze the importance of virtual body presentation in VR on participants’ performances. For each task, we chose specific parameter regarding the quality of performances. We defined a loss in the performance quality if the number of errors and the time for completion increased. In the balance task, we valued a high number of taken foot strikes as a factor of insecurity, and therefore as a further negative impact on performances. Which strategy was chosen to step over the beam is often influenced by the given instructions. Since the instructions were the same, we assumed to see a difference in the behavior or performances of the participants caused by the conditions. To minimize the effect of being in an unfamiliar environment, we included 10 test trials in VR. We were also interested in the subjective estimates of difficulty as well since the impression of the participants could be influenced by not have the feeling of being present, or not feeling of being embodied. In the balance task, the NB condition was observed as the worst compared to the other visualization types (see Fig 3). It could not be shown that an increasing reduction in the visibility of the limbs also leads to an increasing deterioration of the performances. Regarding the number of errors or the time for completion, the performances seemed to improve after removing the feet and legs from vision. Perhaps, for balancing, it is not necessary to fixate the feet or the legs to complete the task. This could also be seen in the throwing task, in which the WB visualization led to the best results through the predefined parameter. This is observable through the reached points in the scoring system. However, in the grasping task seems to be no influence due to the different body visualization types (see Fig 4). Here, the participants’ performances were best in the NB condition regarding the values of the scoring system and the shortest duration of movement execution. In this case, the results suggested that the visualization of the body limbs in VR could be more distractive and led to decreased performances. However, some participants stated that the different types of visualization were often not noticeable due to the limited field of view (FOV) of the HMD in the grasping and throwing task, whereas the visualization of the feet and legs were crucial within the balance task. This could be an explanation for the small number of effects between the different visualization types and is also discussed in limitations and future directions.

Regarding the embodiment, we ensured an enhanced sense of self-location through the first-person-perspective and because of including associated tactile information when the participants have reached out to the ball. Here, the properties of the presented avatar (provided by Vicon) did not fit perfectly for each participant. Although we used a female avatar for a female participant and vice versa, the individual properties of every single body were not reached. When the participant grasped the ball, the fingers were not perfectly hit the surface which could lead to less embodied feeling. The visual-tactile correlations were less compared to the real-world condition. Therefore, a minimal loss of the sense of self-location in the VR must be considered. The sense of agency was provided since the full-body movements of the participants were tracked by using reflective markers, which were attached to the participant´s limbs [12]. Unfortunately, we could not control possible latencies that can occur due to the use of wireless adapted for the HTC Vive Pro. The participants did not recognize any latencies or disruptions of presenting the virtual scene. For the sense of body ownership, the avatars’ appearance was human-like, but, as mentioned before, the morphological similarity between one’s biological body and the virtual one was not provided perfectly. Therefore, the sense of body ownership could have suffered, due to reduced top-down processes, which have a positive aftermath on the perception of ownership of the virtual body. We conclude that the presentation of a realistic whole body without delays and offset is helpful for performance analyses in VR, even for young and healthy participants and with quite easy tasks. Otherwise, the participants need time to get used to it and adjust their performances, which is not reflecting real-world conditions. The main result that has been emerged from the data is that whole-body visualization leads to better results in each tested motoric task than no-body visualization. Overall, the different types of body visualizations seemed to have no significant impact on the participants’ performances. Perhaps, most participants were focused on a specific fixating point in the background during balancing. Therefore, a reduced view of body segments would have no negative influence. Although the application of more technology (in this case the tracking technology for real-time motion capturing of the whole body) often leads to greater problems and higher delays, we had no technical problems. The application of Vicon Shogun and the motion suits (Fig 1) were described as very user-friendly by the participants.

## Limitations und future directions

We need to mention some limitations of the current study. The used HMD had a limited field of view (110°) compared to the eyes in reality (approximately 180°) but this is the case with almost all HMDs. However, that limitation in vision could have led to worse performance in VR compared to reality. The participants often complained that the different visualizations were not noticed at all due to the limited FOV in VR. A larger FOV would solve the problem so that a more realistic impression of the scene could be obtained. In addition, during the balance task, some participants focused on a visual point at the end of the beam. Therefore, they did not even recognize that the feet or legs were not visualized. Additionally, an integrated eye-tracking system could be included to measure participants’ gaze behavior during their performances, since not all participants focused on their limbs during task completion. Furthermore, immersion as a quantifiable aspect of the simulation and the subjective feeling of presence, as well as natural behavior in VR contextual, psychological, personality, and emotional aspects are also very important [13]. However, we did not include further questionnaires to assess further aspects, such as presence or cybersickness. Our participants told us later that they had no problems with cybersickness (at exposure time to VR of around 30 minutes) and they rated the VR and the virtual body to be realistic.

An additional factor that limits the current study is the measure of the presence or the experiences of body ownership [49]. The focus was to analyze the performances during the different motoric tasks and to compare them between the conditions. Established questionnaires could have been used to amplify the knowledge about the impact of different body visualization types. Also, it is recommended to measure physiological responses and behaviors, which may indicate whether the participants felt that they were in the scenario [12]. The results suggest that an impact on the feeling of presence just occurred when no-body visualization was used since the quality of participants’ performances decreased.

Although we randomized the body presentations in all tasks, the “no-body conditions” occurred in the first half of the 12 repetitions. Thus, the order could have affected our results. Moreover, when occluding the whole body, which is a very unfamiliar situation, participants need to rely more on body information (proprioception and muscle sensations). On the one hand, that situation can be used for training to trust more on own body information, but on the other hand, maybe the familiarization phase (one minute watching the VR scene and ten times walking over the beam in whole-body condition in VR) were too short.

We did not analyze the performance with different types of virtual bodies in VR. We only provided a virtual body (with male and female properties) of the same height as the tested person but the shape was similar for each participant. Both in reality and in VR, the participants wore a black motion suit with the attached markers, and in VR, they saw a black-dressed avatar (Fig 1). In future studies, it should be analyzed if the performance changes when the avatar (as the own body) is even modeled closer to the own real body related to the visual-tactile, visual-proprioceptive and visual-motoric matching.

We are not able to derive further recommendations for different tested groups or athletes of different ages, gender, and sports. We could show that the methods used in the current study are appropriate to analyze performance and behavior in VR and reality. The sports-related tasks are doable for young and healthy adults. Although they seem to be quite easy in reality, we found significant differences between VR and reality. However, the subjective estimation was only low and moderate for the tasks in both conditions. Therefore, it would be interesting to repeat our study with different participant groups (e.g. different ages, different sports backgrounds, or athletes of different expertise levels) and to further include more tasks, which could also be more sports specific.

Another interesting aspect would be the transfer from VR to RW. If the quality of the movements, which were only learned or performed in VR, can be transferred to the real environment, it can be assumed that the perception from VR is the same as in the real one.

## Conclusion

The current study is the first study comparing sports-related tasks in VR and in reality with further manipulations (occlusions of body parts) of the virtual body visualization. Realistic virtual environments and objects were provided and natural movements were allowed. Due to the lack of haptic feedback in VR, we gave the participants a real ball for grasping and throwing and a real balance beam, which were virtualized in real-time. Thus, realistic conditions were ensured. However, significant differences in the performance in all tasks were found between reality and VR, especially in the time of completion and the subjective estimation of difficulty. Moreover, the results show that the whole-body visualization leads to the best performances (lower time of completion, number of foot strikes during the balance task) in contrast to the other visualization types, especially for the no-body condition. We conclude that the visualization of a realistic virtual body is helpful to limit differences between both conditions and to ensure quite natural body perception. For task completion, however, it is not always necessary to visualize the whole-body, since no differences in performances could be detected for the reduced vision of body limbs. For studies analyzing perception and sports performance or for VR sports interventions, we recommend at least the visualization of the task-specific body parts, such as during throwing the hands and arms or during balancing the feet and legs in real-time. Besides, a further experiment may provide information on whether the participants used the incoming visual information in VR to complete the balance task. We observed habituation on performances, especially during the balance task. Therefore, we concluded to let the participants walk blindfolded over the beam to exclude visual information. Probably it is still possible to succeed due to haptic feedback, which would lead to similar quality in performances compared to VR conditions.
